# CeModule: an integrative framework for discovering regulatory patterns from genomic data in cancer

**DOI:** 10.1186/s12859-019-2654-3

**Published:** 2019-02-07

**Authors:** Qiu Xiao, Jiawei Luo, Cheng Liang, Jie Cai, Guanghui Li, Buwen Cao

**Affiliations:** 1 College of Computer Science and Electronic Engineering, Hunan University, Changsha, 410082 China; 2 Hunan Provincial Key Laboratory of Intelligent Computing and Language Information Processing, Hunan Normal University, Changsha, 410081 China; 3 College of Information Science and Engineering, Shandong Normal University, Jinan, 250000 China

**Keywords:** Regulatory pattern, Module discovery, microRNA, lncRNA function, ceRNA, Cancer, Machine learning

## Abstract

**Background:**

Non-coding RNAs (ncRNAs) are emerging as key regulators and play critical roles in a wide range of tumorigenesis. Recent studies have suggested that long non-coding RNAs (lncRNAs) could interact with microRNAs (miRNAs) and indirectly regulate miRNA targets through competing interactions. Therefore, uncovering the competing endogenous RNA (ceRNA) regulatory mechanism of lncRNAs, miRNAs and mRNAs in post-transcriptional level will aid in deciphering the underlying pathogenesis of human polygenic diseases and may unveil new diagnostic and therapeutic opportunities. However, the functional roles of vast majority of cancer specific ncRNAs and their combinational regulation patterns are still insufficiently understood.

**Results:**

Here we develop an integrative framework called CeModule to discover lncRNA, miRNA and mRNA-associated regulatory modules. We fully utilize the matched expression profiles of lncRNAs, miRNAs and mRNAs and establish a model based on joint orthogonality non-negative matrix factorization for identifying modules. Meanwhile, we impose the experimentally verified miRNA-lncRNA interactions, the validated miRNA-mRNA interactions and the weighted gene-gene network into this framework to improve the module accuracy through the network-based penalties. The sparse regularizations are also used to help this model obtain modular sparse solutions. Finally, an iterative multiplicative updating algorithm is adopted to solve the optimization problem.

**Conclusions:**

We applied CeModule to two cancer datasets including ovarian cancer (OV) and uterine corpus endometrial carcinoma (UCEC) obtained from TCGA. The modular analysis indicated that the identified modules involving lncRNAs, miRNAs and mRNAs are significantly associated and functionally enriched in cancer-related biological processes and pathways, which may provide new insights into the complex regulatory mechanism of human diseases at the system level.

**Electronic supplementary material:**

The online version of this article (10.1186/s12859-019-2654-3) contains supplementary material, which is available to authorized users.

## Background

MicroRNAs (miRNAs) are small (~ 22 nt), endogenous, single-stranded and non-coding RNA molecules, which play crucial roles in post-transcriptional regulation by repressing mRNA translation or destabilizing target mRNAs [1]. Many studies have revealed that the mutation and dysregulated miRNA expression may cause various human diseases [2, 3]. MiRNAs act as essential components of complex regulatory networks and are involved in many different biological processes, such as cell proliferation, metabolism, and oncogenesis [4–6]. Therefore, understanding the functional roles and regulatory mechanisms of miRNAs will greatly facilitate the diagnosis and treatment of human diseases [7, 8].

Recently, a competing endogenous RNA (ceRNA) hypothesis has been presented by Salmena et al. [9], which has dramatically shifted our understanding of miRNA regulatory mechanism. The complex ceRNA post-transcriptional regulatory mechanism reported that by sharing common miRNA response elements (MREs), several types of competing endogenous RNAs or miRNA sponges (e.g. lncRNAs, pseudogenes and circRNAs) compete with protein-coding RNAs for binding to miRNAs, thereby relieving miRNA-mediated target repression. Numerous convincing evidence has been discovered in a variety of species by biological experiments [10, 11]. For example, the study found that lncRNA HULS plays an important role in liver cancer, which serves as an endogenous sponge by reducing miR-372-mediated translational repression of PRKACB [12]. IPS1 overexpression has also been reported to increase the expression of PHO2 by competitively interacting with miR-399 in arabidopsis [13]. In addition, numerous studies have shown that ceRNA crosstalk exists in a variety of cellular behaviors, and many diseases are affected by their disturbances [14, 15]. However, the cooperative regulation mechanisms and the roles of ceRNA–associated activities in physiologic and pathologic conditions are in their infancy, and thus require further research.

The development of high-throughput techniques has made a vast amount of omics data to be publicly available, thereby enabling systematic investigation of the complex regulatory networks. Great efforts have been made to decipher the interaction mechanism of numerous biomolecules in a transcriptional or post-transcriptional level, such as co-regulatory motif discovery [16], miRNA-mRNA regulatory module identification [17, 18], miRNA and TF (transcription factor) co-regulation inference [19]. Meanwhile, other methods have been developed to prioritize cancer-related biological molecules, such as miRNAs [20, 21]. Undoubtedly, all these studies provide a global perspective for the study of combinatorial effects and human complex diseases.

In recent years, lncRNAs as a class of ncRNAs and miRNA sponges have been identified in many human cancers [22]. Some systematic studies on many diseases have been carried out [23–25]. In addition, some tools related to lncRNA, such as DIANA-LncBase [26], Linc2GO [27] and LncRNADisease [28], have been developed. However, the functions and modular organizations of most of lncRNAs are still not clear, and the novel regulatory mechanism based on ceRNA hypothesis requires comprehensive investigation. To the best of our knowledge, little effort has been devoted to methods that are specifically designed to investigate the cancer-specific regulatory patterns involved in miRNA and miRNA sponges on a large scale.

In this study, we develop a novel integrative framework called CeModule to systematically detect regulatory patterns involving lncRNAs, miRNAs, and mRNAs. The proposed method fully exploits the lncRNA/miRNA/mRNA expression profiles, the experimentally determined miRNA-lncRNA interactions, the verified miRNA-mRNA interactions, and the weighted gene-gene functional interactions. Here, inspired by [29–31], we adopt a model with joint orthogonality non-negative matrix factorization to detect these modules. In addition, both network-regularized constraints and sparsity penalties are incorporated into the model for helping to discover and characteriz the lncRNA-miRNA-mRNA associated regulatory modules. Finally, we apply the proposed method to ovarian cancer (OV) and uterine corpus endometrial carcinoma (UCEC) datasets downloaded from TCGA [32]. The results indicate that CeModule could be effectively applied to the discovery of biologically function modules, which greatly advances our understanding of the coordination mechanisms on a system level.

## Methods

In the following sections, we will first introduce the mathematical formulation of CeModule. Afterwards, the modules are identified based on the decomposed matrix components. Finally, several experiments and literature surveys are performed to systematically evaluate these modules.

### The CeModule algorithm for identifying modules by integrating massive genomic data

#### Joint orthogonal non-negative matrix factorization

In this study, we identify the lncRNA, miRNA and mRNA-associated regulatory modules by a non-negative matrix factorization (NMF)-based framework. The corresponding objective function of standard NMF [31, 33] is formulated as follows:

1 $$ \underset{W,H}{\min }{\left\Vert X-{WH}^T\right\Vert}_F^2s.t.\kern0.5em W\ge 0,H\ge 0 $$

where ||.||_*F*_ denotes the Frobenius norm.

Existing studies have indicated that orthogonality NMF could produce a better modularity interpretation [6, 30, 34]. Therefore, we present a integrative framework using joint orthogonality NMF to determine the module regulation and membership through simultaneously integrating multiple data sources. To clearly describe the problem, let *X*_*1*_∈*R* ^*S × N1*^, *X*_*2*_∈*R*^*S × N2*,^ and *X*_*3*_∈*R*^*S × N3*^ denote the lncRNA, miRNA, and mRNA expression matrices, respectively. Subsequently, we define an objective function of joint orthogonality NMF as follows:

2 $$ {\displaystyle \begin{array}{c}\underset{W,{H}_1,{H}_2,H3}{\min}\sum \limits_{i=1,2,3}\left(\left\Vert {X}_i-{WH}_i^T\left\Vert {}_F^2+\frac{1}{2}\alpha \left\Vert {H}_i^T{H}_i-I\left\Vert {}_F^2\right.\right.\right.\right.\right)\\ {}s.t\kern0.5em W\ge 0,{H}_i\ge 0\end{array}} $$

where *W*(size:*S* × *K*) denotes the common basic matrix; coefficient matrices *H*_*1*_, *H*_*2*_, and *H*_*3*_ have dimensions *N1* × *K*, *N2* × *K*, and *N3* × *K*, respectively; *α* is the hyperparameter that controls the trade-off of *H*_*i*_.; dimension *K* represents the desired number of modules.

However, many data sources often contain noise, and several investigations of NMF have been conducted to improve the performance [35]. To obtain sparse solutions and regulatory modules with better biological interpretation, the sparse constraints were incorporated into this model similar to that suggested by Hoyer [36], which can effectively make matrices *H*_*i*_ sparse. The objective function of joint orthogonality NMF with sparsity penalties can be written as follows:

3 $$ {\displaystyle \begin{array}{c}\underset{W,{H}_1,{H}_2,{H}_3}{\min}\sum \limits_{i=1,2,3}\left({\left\Vert {X}_i-{WH_i}^T\right\Vert}_F^2+\frac{1}{2}\alpha {\left\Vert {H_i}^T{H}_i-I\right\Vert}_F^2\right)\\ {}+{\gamma}_1{\left\Vert W\right\Vert}_F^2+{\gamma}_2\sum \limits_{i=1,2,3}{\left\Vert {H}_i\right\Vert}_1\\ {}s.t.\kern0.5em W\ge 0,{H}_i\ge 0\end{array}} $$

where *γ*_*1*_ and *γ*_*2*_ are the regularization coefficients.

#### The mathematical formulation of CeModule

Apart from the expression profiles, the data sources including miRNA-lncRNA interactions, miRNA-mRNA interactions and gene-gene network have also been fully utilized to improve the performance. Here, to improve the quality of identified modules, the network-based penalties are imposed on this computational model based on Hoyer’s work [6, 36] and make sure that those tightly linked lncRNAs/miRNAs/mRNAs are forced to assign into the same module.

Let *A*∈*R*^*N2 × N1*^ and *B*∈*R*^*N2 × N3*^ denote the adjacency matrices of miRNA-lncRNA and miRNA-mRNA interaction networks, respectively, *C*∈*R*^*N3 × N3*^ is the matrix of gene-gene functional interaction network. For the miRNA-lncRNA interaction network, we perform the network-based constraints according to the objective function as follows:

4 $$ {O}_1=\sum \limits_{ij}{a}_{ij}{h_i}^{(2)}{\left({h_j}^{(1)}\right)}^T= Tr\left({H_2}^T{AH}_1\right) $$

where *a*_*ij*_ is the entity of *A*; *h*_*i*_^*(2)*^ and *h*_*j*_^*(1)*^ represent the *i*th and *j*th rows of *H*_*2*_ and *H*_*1*_, respectively. Similarly, the corresponding objective functions of two other networks can be obtained as follows:

5 $$ {O}_2=\sum \limits_{ij}{b}_{ij}{h_i}^{(2)}{\left({h_j}^{(3)}\right)}^T= Tr\left({H_2}^T{BH}_3\right) $$

6 $$ {O}_3=\sum \limits_{ij}{c}_{ij}{h_i}^{(3)}{\left({h_j}^{(3)}\right)}^T= Tr\left({H_3}^T{CH}_3\right) $$

Then, combining the function in Eq. (3) with three network-based regularization terms, we can mathematically formulate the optimization problem of CeModule as follows:

7 $$ {\displaystyle \begin{array}{c}\underset{W,{H}_1,{H}_2,{H}_3}{\min}\sum \limits_{i=1,2,3}\left({\left\Vert {X}_i-{WH_i}^T\right\Vert}_F^2+\frac{1}{2}\alpha {\left\Vert {H_i}^T{H}_i-I\right\Vert}_F^2\right)\\ {}-{\lambda}_1 Tr\left({H_2}^T{AH}_1\right)-{\lambda}_2 Tr\left({H_2}^T{BH}_3\right)-{\lambda}_3 Tr\left({H_3}^T{CH}_3\right)\\ {}+{\gamma}_1{\left\Vert W\right\Vert}_F^2+{\gamma}_2\sum \limits_{i=1,2,3}{\left\Vert {H}_i\right\Vert}_1\\ {}s.t.\kern0.5em W\ge 0,{H}_i\ge 0\end{array}} $$

where λ_*1*_, λ_*2*_ and λ_*3*_ are the regularization parameters. In the following, we adopt an iterative updating method [37] to obtain local optimal solution for the optimization problem.

Let *Φ* = [*φ*_*lk*_],*Ψ* = [*ψ*_*jk*_], *Ω =* [*ω*_*pk*_], and *Θ* = [*θ*_*qk*_] be the Lagrange multipliers for constrain *w*_*lk*_ ≥ 0, *h*_*jk*_^*(1)*^ ≥ 0, *h*_*pk*_^*(2)*^ ≥ 0, and *h*_*pk*_^*(3)*^ ≥ 0, respectively. We can obtain the Lagrange function of Eq. (7) as follows:

8 $$ {\displaystyle \begin{array}{c}{L}_f={\sum}_{i=1}^3\left[ Tr\left({X}_i{X_i}^T\right)-2 Tr\left({X}_i{H}_i{W}^T\right)+ Tr\left({WH_i}^T{H}_i{W}^T\right)\right.\\ {}\left.+\frac{1}{2}\alpha \left( Tr\left({H_i}^T{H}_i{H_i}^T{H}_i\right)-2 Tr\left({H_i}^T{H}_i\right)+ Tr\left({I}^TI\right)\right)\right]\\ {}-{\lambda}_1 Tr\left({H_2}^T{AH}_1\right)-{\lambda}_2 Tr\left({H_2}^T{BH}_3\right)-{\lambda}_3 Tr\left({H_3}^T{CH}_3\right)\\ {}+{\gamma}_1 Tr\left({WW}^T\right)+{\gamma}_2\sum \limits_{i=1}^3 Tr\left({E_i}^T{H}_i\right)\\ {}+ Tr\left(\Phi {W}^T\right)+ Tr\left(\Psi {H_1}^T\right)+ Tr\left(\Omega {H_2}^T\right)+ Tr\left(\Theta {H_3}^T\right)\end{array}} $$

where *E*_*1*_∈{1}^*N1 × K*^, *E*_*2*_∈{1}^*N2 × K*^, and *E*_*3*_∈{1}^*N3 × K*^. The partial derivatives of the above function for *W* and *H*_*i*_ are:

9 $$ {\displaystyle \begin{array}{c}\frac{\partial {L}_f}{\partial W}={\sum}_{i=1}^3\left[-2{X}_i{H}_i+2{WH_i}^T{H}_i\right]+2{\gamma}_1W+\Phi \\ {}\frac{\partial {L}_f}{\partial {H}_1}=-2{X_1}^TW+2{H}_1{W}^TW+\frac{1}{2}\alpha \left(4{H}_1{H_1}^T{H}_1-4{H}_1\right)\\ {}-{\lambda}_1{A}^T{H}_2+{\gamma}_2{E}_1+\Psi \\ {}\frac{\partial {L}_f}{\partial {H}_2}=-2{X_2}^TW+2{H}_2{W}^TW+\frac{1}{2}\alpha \left(4{H}_2{H_2}^T{H}_2-4{H}_2\right)\\ {}-{\lambda}_1{AH}_1-{\lambda}_2{BH}_3+{\gamma}_2{E}_2+\Omega \\ {}\frac{\partial {L}_f}{\partial {H}_3}=-2{X_3}^TW+2{H}_3{W}^TW+\frac{1}{2}\alpha \left(4{H}_3{H_3}^T{H}_3-4{H}_3\right)\\ {}-{\lambda}_2{B}^T{H}_2-2{\lambda}_3{CH}_3+{\gamma}_2{E}_3+\Theta \end{array}} $$

Using the KKT conditions [38, 39] *φ*_*lk*_*w*_*lk*_ = 0, *ψ*_*jk*_*h*_*jk*_^*(1)*^ = 0, *ω*_*pk*_*h*_*pk*_^*(2)*^ = 0, and *θ*_*qk*_*h*_*pk*_^*(3)*^ = 0, we obtain the following equations for *w*_*lk*_, *h*_*jk*_^*(1)*^, *h*_*pk*_^*(2)*^, and *h*_*pk*_^*(3)*^:

10 $$ {\displaystyle \begin{array}{c}-2\sum \limits_{i=1}^3{\left({X}_i{H}_i\right)}_{lk}{w}_{lk}+2{\left[{\sum}_{i=1}^3\left({WH_i}^T{H}_i\right)+\left({\gamma}_1W\right)\right]}_{ik}{w}_{lk}=0\\ {}{\left(-2{X_1}^TW-2\alpha {H}_1-{\lambda}_1{A}^T{H}_2\right)}_{jk}{h}_{jk}^{(1)}\\ {}+{\left(2{H}_1{W}^TW+2\alpha {H}_1{H_1}^T{H}_1+{\gamma}_2{E}_1\right)}_{jk}{h}_{jk}^{(1)}=0\\ {}{\left(-2{X_2}^TW-2\alpha {H}_2-{\lambda}_1{AH}_1-{\lambda}_2{BH}_3\right)}_{pk}{h}_{pk}^{(2)}\\ {}+{\left(2{H}_2{W}^TW+2\alpha {H}_2{H_2}^T{H}_2+{\gamma}_2{E}_2\right)}_{pk}{h}_{pk}^{(2)}=0\\ {}{\left(-2{X_3}^TW-2\alpha {H}_3-{\lambda}_2{B}^T{H}_2-2{\lambda}_3{CH}_3\right)}_{qk}{h}_{qk}^{(3)}\\ {}+{\left(2{H}_3{W}^TW+2\alpha {H}_3{H_3}^T{H}_3+{\gamma}_2{E}_3\right)}_{qk}{h}_{qk}^{(3)}=0\end{array}} $$

Finally, we determine the multiplicative update rules for *W* and *H*_*i*_ as follows:

11 $$ {\displaystyle \begin{array}{c}{w}_{lk}\leftarrow {w}_{lk}\frac{{\left({X}_1{H}_1+{X}_2{H}_2+{X}_3{H}_3\right)}_{lk}}{{\left({WH_1}^T{H}_1+{WH_2}^T{H}_2+{WH_3}^T{H}_3+{\gamma}_1W\right)}_{lk}}\\ {}{h}_{jk}^{(1)}\leftarrow {h}_{jk}^{(1)}\frac{{\left({X_1}^TW+\alpha {H}_1+\frac{\lambda_1}{2}{A}^T{H}_2\right)}_{jk}}{{\left({H}_1{W}^TW+\alpha {H}_1{H_1}^T{H}_1+\frac{\gamma_2}{2}{E}_1\right)}_{jk}}\\ {}{h}_{pk}^{(2)}\leftarrow {h}_{pk}^{(2)}\frac{{\left({X_2}^TW+\alpha {H}_2+\frac{\lambda_1}{2}{AH}_1+\frac{\lambda_2}{2}{BH}_3\right)}_{pk}}{{\left({H}_2{W}^TW+\alpha {H}_2{H_2}^T{H}_2+\frac{\gamma_2}{2}{E}_2\right)}_{pk}}\\ {}{h}_{qk}^{(3)}\leftarrow {h}_{qk}^{(3)}\frac{{\left({X_3}^TW+\alpha {H}_3+\frac{\lambda_2}{2}{B}^T{H}_2+{\lambda}_3{CH}_3\right)}_{qk}}{{\left({H}_3{W}^TW+\alpha {H}_3{H_3}^T{H}_3+\frac{\gamma_2}{2}{E}_3\right)}_{qk}}\end{array}} $$

The four non-negative matrices *W*, *H*_*1*_, *H*_*2*_ and *H*_*3*_ are updated according to the above rules until convergence. More details about the derivations and proof for the convergence of the optimization problem are provided in the Additional file 1.

#### Determining ceRNA modules

**Fig. 1:**
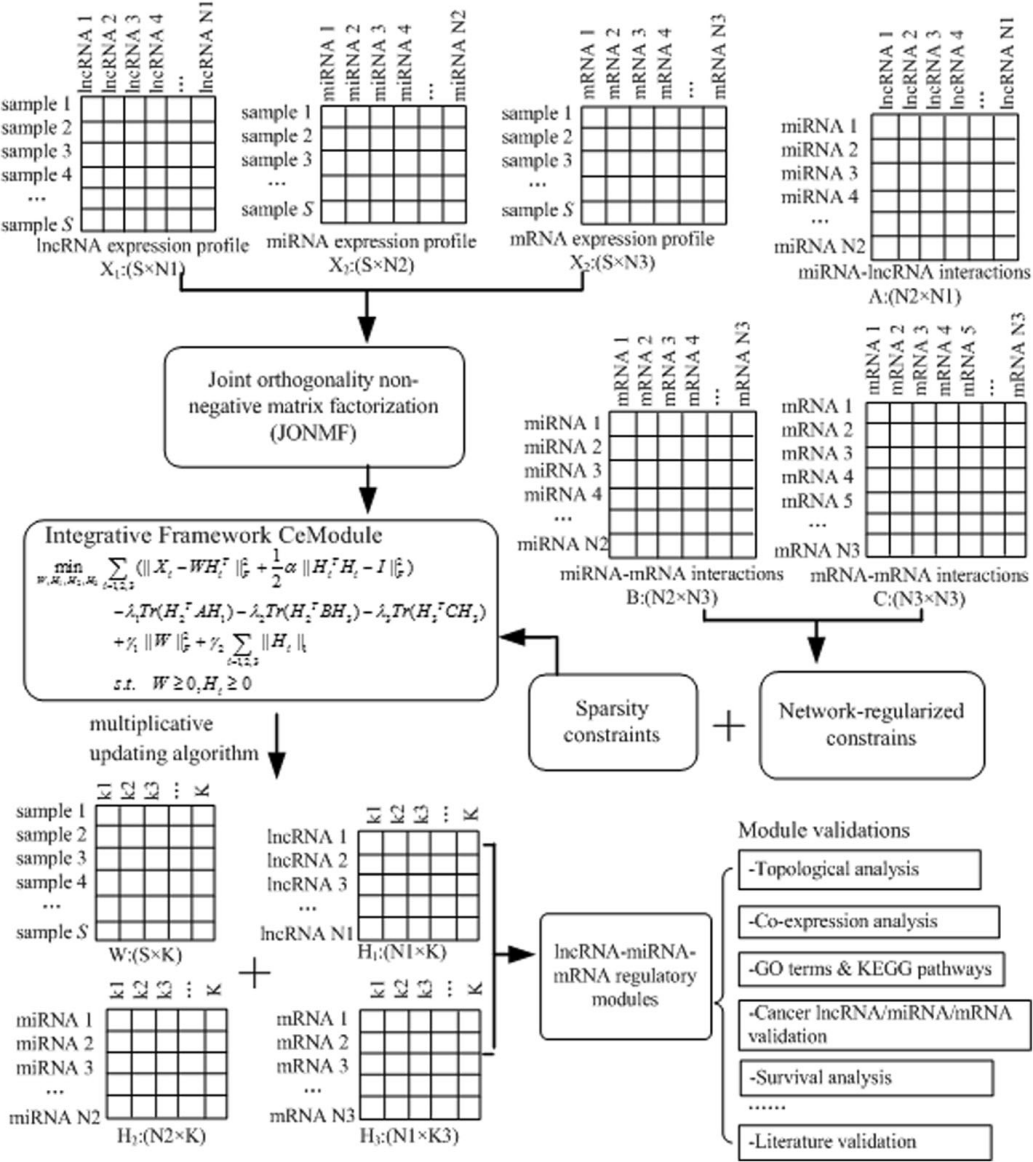
Overall workflow of CeModule for detecting lncRNA, miRNA, and mRNA-associated regulatory patterns

The obtained coefficient matrices *H*_*1*_, *H*_*2*_, and *H*_*3*_ will guide us to detect ceRNA-associated regulatory modules. Here, similar to the way for identifying co-modules developed by Chen et al. [40], we obtain a z-score for each element based on the columns of *H*_*1*_, *H*_*2*_, and *H*_*3*_ as follows: *z*_*ij*_ = (*x*_*ij*_-μ_j_)/*σ*_*j*_, where *μ*_*j*_ denotes the average value of lncRNA (or miRNA, mRNA) *i* in *H*_*1*_ (or *H*_*2*_, *H*_*3*_), and *σ*_*j*_ is the standard deviation. Subsequently, we assign lncRNA (or miRNA, mRNA) *i* into module *j* if *z*_*ij*_ exceeds a given threshold *T*, and then all the ceRNA-associated modules can be obtained. The overall workflow of the proposed CeModule framework for identifying regulatory module is shown in Fig. 1.

### Experimental setup and module validation

We systematically evaluate the performance of CeModule by conducting a functional enrichment analysis for genes in each module. We downloaded the GO (Gene Ontology) terms in biological process from http://www.geneontology.org/, and obtained the canonical pathways from MSigDB [41]. We removed the GO terms with evidence codes equal to NAS (Non-traceable Author Statement), ND (No biological Data available) or EA (Electronic Annotation) and those with fewer than 5 genes similar to Li et al. [18]. The hypergeometric test was used to calculate the statistical significance for genes in each module with respect to each GO term or pathway. Meanwhile, we used TAM [42], which is a free online tool for annotations of human miRNAs, to perform enrichment analysis for miRNAs in the identified modules.

We also investigate the miRNA cluster/family enrichment for each module, and obtained the miRNA cluster information and miRNA families from miRBase (http://www.mirbase.org/) (release 21) [43]. Furthermore, to determine whether these modules related to specific cancer, we acquired those known cancer-related lncRNAs from LncRNADisease [28] and Lnc2Cancer [44]. The verified disease-related miRNAs and genes were collected from HMDD v2.0 [45], and DisGeNET [46], respectively.

Additionally, the method contains several parameters, more detailed information about them are illustrated in Additional file 1. Here, we determined the values of reduced dimension *K* on the basis of a miRNA cluster analysis. The results show that the miRNAs used in this study covered 69/76 miRNA clusters with an average of about 2.7/2.3 miRNAs per cluster for OV/UCEC dataset. Therefore, we set *K* to 70 in the two cancer datasets, which is approximately equal to the number of miRNA clusters.

## Results

### Data sources and preprocessing

We applied CeModule to ovarian cancer (OV) and uterine corpus endometrial carcinoma (UCEC) genomic data and downloaded the matched mRNA and lncRNA expression profiles from http://www.larssonlab.org/tcga-lncrnas/ [47]. Due to the expression values of many lncRNAs/mRNAs in the original data source are all zeros or close to zeros, as done in [48], we removed some lncRNAs/mRNAs in the expression profiles with a variance less than the percentile specified by a cutoff (30%) and filter those lncRNAs/mRNAs with overall small absolute values less than another percentile cutoff (60%). The corresponding Matlab functions are *genevarfilter* and *genelowvalfilter*, respectively. We obtained the miRNA expression profiles of OV/UCEC from the TCGA data portal (http://cancergenome.nih.gov/) and removed the rows (or miRNAs) where all the expression values are zeros. These expression data were further log2-transformed. Finally, the datasets contain 7982(8056) lncRNAs, 415(505) miRNAs, and 10,618(10308) mRNAs across 385(183) matched samples for OV (UCEC), which were represented in three matrices *X*_*1*_, *X*_*2*_ and *X*_*3*_, and then the method in [49] is adopted to ensure non-negative constraints.

The experimentally verified interactions between miRNAs and lncRNAs were downloaded from DIANA-LncBase [26] and starBase v2.0 [50]. We obtained the miRNA targets from three experimentally verified databases, including miRecords (version 4.0) [51], TarBase (version 6.0) [52], and miRTarBase (version 6.1) [53]. After filtering out duplicate interactions or interactions involving lncRNAs, miRNAs, and mRNAs that were absent in the expression profiles, 12,969/6165 miRNA-lncRNA and 20,848/25447 miRNA-mRNA interactions were finally retained for OV/UCEC dataset. The weighted gene-gene network is derived from HumanNet [54], which is a probabilistic functional gene network. After filtering those genes absent from the expression data, 536,698/252021 interactions are retained for OV/UCEC. Finally, we obtained the miRNA-lncRNA matrix *A*, the miRNA-mRNA matrix *B* and the gene-gene matrix *C*.

#### Topological characteristics analysis

We identified modules in ovarian cancer and uterine corpus endometrial carcinoma by integrating multiple heterogeneous data sources, and obtained 70 modules for OV/UCEC (Additional file 2: Table S1) with an average of 68.2/46.1 lncRNAs, 6.3/5.5 miRNAs, and 55.5/48.1 mRNAs per module. The distributions of number of lncRNAs, miRNAs, and mRNAs for the identified modules for OV and UCEC datasets are displayed in Additional file 1: Figure S1 and S2.

**Fig. 2:**
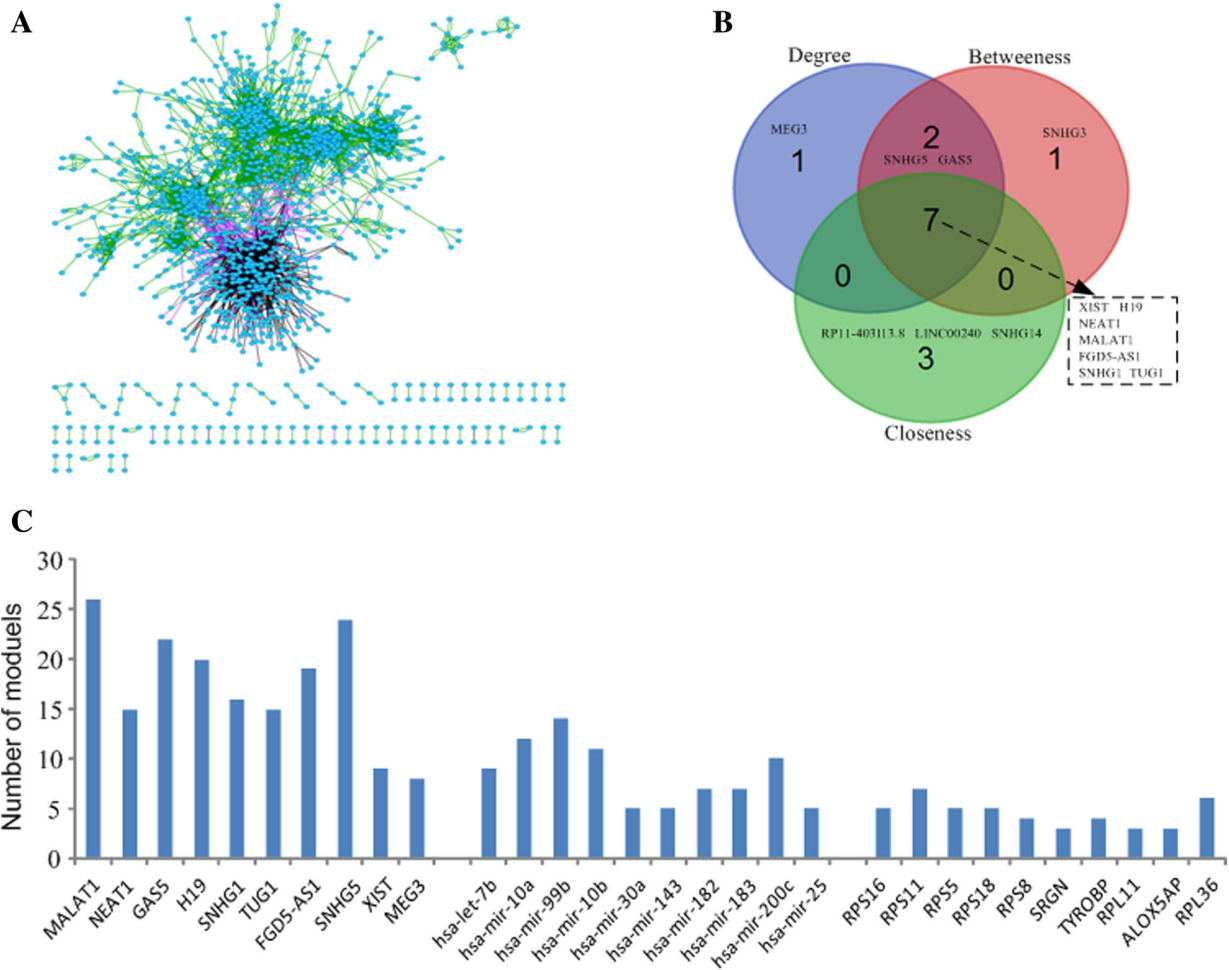
Topological features of the identified modules and the ceRNA regulatory network for ovarian cancer. **a** View of the ceRNA module network in OV. If two nodes are members of a module and their interactions exist in the databases as mentioned in the aforementioned interaction databases, then an edge between the two nodes is displayed. Three colors (black, purple and green) correspond to three types of interactions (lncRNA-miRNA, miRNA-gene and gene-gene). Nodes with no edges are omitted to improve visualization. **b** Overlap of the top 10 lncRNAs across three dimensions for OV. **c** The distributions of number of modules identified by CeModule for the top 10 lncRNAs, miRNAs, and mRNAs with the highest degree in OV dataset

According to the constructed regulatory networks by merging those modules identified by our method, we found that a small number of nodes are more likely to be hubs or act as bridges, and tend to be involved in more competing interactions and participate in more human diseases. For instance, Fig. 2a presents a global view of the regulatory network for OV, which demonstrated that the network was densely connected and a small fraction of the nodes presented significantly higher degree, betweenness centrality, and closeness centrality than other nodes. The top 10 lncRNAs/miRNAs/mRNAs for each dimension (degree, closeness, and betweenness) in the networks of OV and UCEC datasets are listed in Table 1 and Additional file 1: Table S2, and there are substantial overlaps exist across the three dimensions (Fig. 2b and Additional file 1: Figure S3 and S4). Meanwhile, as shown in Fig. 2c and Additional file 1: Table S2, we found that all the top 10 lncRNAs (MALAT1, NEAT1, GAS5, H19, SNHG1, TUG1, FGD5-AS1, SNHG5, XIST, MEG3) and 8 out of the top 10 lncRNAs (MAL2, XIST, SCAMP1, C17orf76-AS1, MALAT1, C11orf95, SEC22B, UBXN8) with the highest degree participate in at least 5 or more modules in OV and UCEC datasets, respectively. The number distributions of modules for all the module members (lncRNAs/miRNAs/mRNAs) are provided in Additional file 2: Table S1.

**Table 1:** The top 10 lncRNAs, miRNAs and mRNAs with the highest degree, closeness centrality, and betweenness centrality in OV

Rank	Degree			Betweenness			Closeness		
	lncRNAs	miRNAs	mRNAs	lncRNAs	miRNAs	mRNAs	lncRNAs	miRNAs	mRNAs
1	MALAT1	let-7b	RPS16	MALAT1	mir-10a	TCF7L1	LINC00240	mir-155	NME5
2	NEAT1	mir-10a	RPS11	NEAT1	let-7b	SNRPF	RP11-403I13.8	mir-506	HIF3A
3	GAS5	mir-99b	RPS5	H19	mir-30a	PTP4A3	MALAT1	mir-206	TCF7L1
4	H19	mir-10b	RPS18	GAS5	mir-146a	PRRX2	NEAT1	mir-223	LRRC6
5	SNHG1	mir-30a	RPS8	TUG1	mir-375	PNISR	FGD5-AS1	mir-10a	ACTG1
6	TUG1	mir-143	SRGN	FGD5-AS1	mir-149	NR5A1	H19	mir-30a	PNISR
7	FGD5-AS1	mir-182	TYROBP	SNHG5	mir-99b	LRRC6	TUG1	let-7b	PRRX2
8	SNHG5	mir-183	RPL11	XIST	mir-183	HIF3A	XIST	mir-197	PTP4A3
9	XIST	mir-200c	ALOX5AP	SNHG1	mir-143	CTSD	SNHG1	mir-146a	CTSD
10	MEG3	mir-25	RPL3	SNHG3	mir-320a	ACTG1	SNHG14	mir-25	SNRPF

**Fig. 3:**
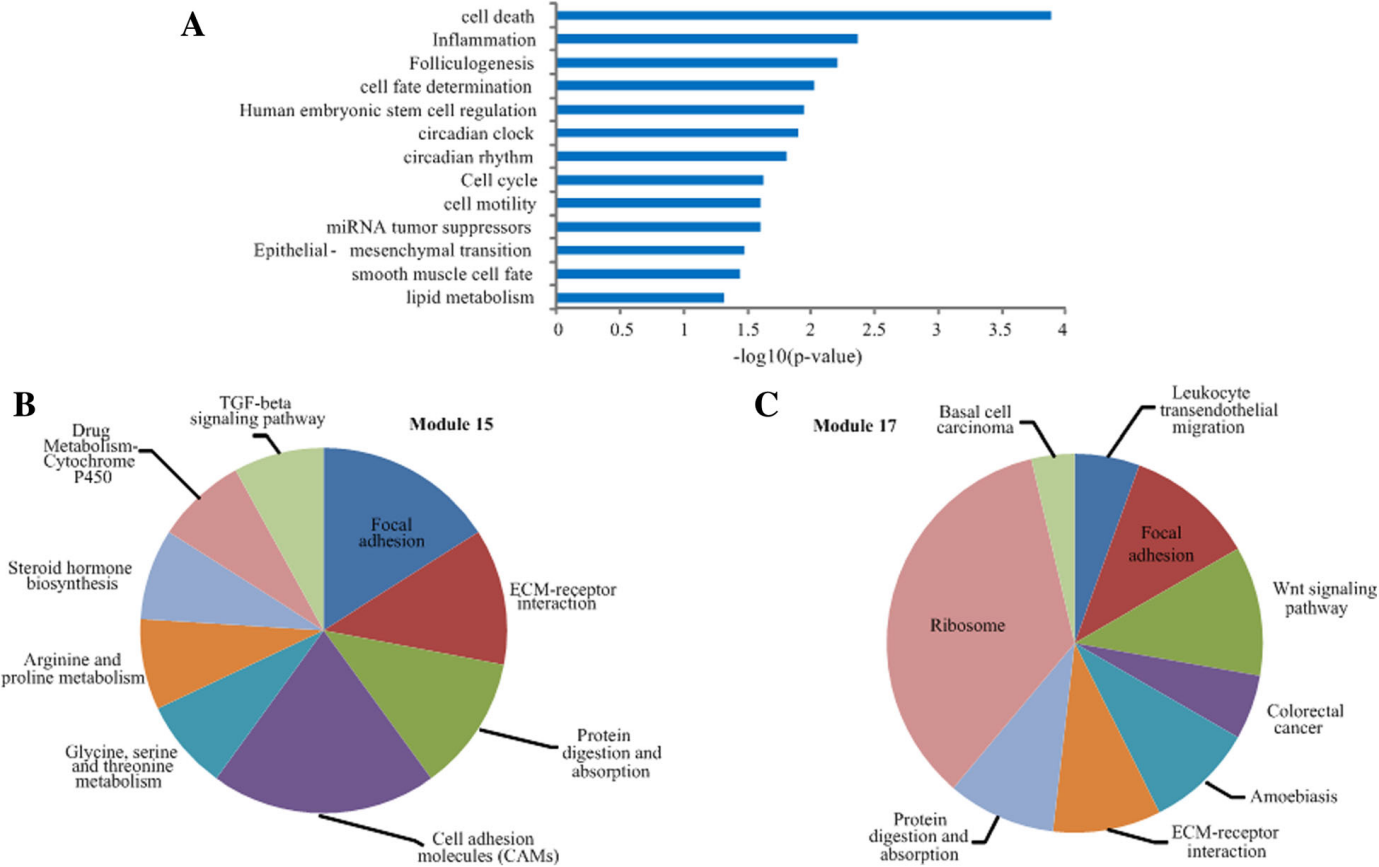
**a** Functional enrichment analysis for the 10 miRNAs with the highest degree using TAM in OV. **b** Pathway enrichment analysis of the module 15 in OV dataset. **c** Pathway enrichment analysis of the module 17 in OV dataset. The area proportion of each pathway presents the number of genes enriched in this pathway

On the other hand, most of the above lncRNAs are supported to be associated with different cancers by public databases or literature. For example, MALAT1 was found to be overexpressed in many solid tumors such as hepatocellular carcinoma [55] and lung cancer [56]. The downregulation of MEG3 is related to poor prognosis and promotes cell proliferation in gastric cancer [57] and bladder cancer [58]. Moreover, MALAT1, NEAT1, GAS5, H19 and XIST have been experimentally validated to be ovarian cancer-related lncRNAs [44], which were identified as hubs that connect 26, 15, 22, 20 and 9 modules in OV dataset, respectively. Additionally, MALAT1 also has been supported to be related to uterine corpus endometrial carcinoma and connected 7 modules in UCEC dataset. The above observations indicate that these lncRNAs can control communication among different functional components in the two datasets. Meanwhile, 8 (let-7b, mir-99b, mir-10b, mir-30a, mir-182, mir-183, mir-200c, mir-25) and 5 (mir-141, mir-10a, mir-200a, let-7b, mir-200b) of the 10 miRNAs with the highest degree are confirmed to be the well-known OV-related and UCEC-related miRNAs by HMDD [45]. We also found that these miRNAs are significantly enriched in cell cycle-related biological processes (Fig. 3a). In addition, we performed the same analysis for mRNAs and also came to the similar observations.

### Functional enrichments of modules

To investigate the functional significance of the identified modules in ovarian cancer and uterine corpus endometrial carcinoma datasets, we perform GO biological process and KEGG pathway enrichment analyses using hypergeometric test for coding genes in each of the modules (FDR < 0.05). The enriched GO terms and KEGG pathways of all the identified modules for OV and UCEC datasets are listed in Additional file 3: Table S3 and Additional file 4: Table S4. The results show that about 88.6%/91.4% of the modules in OV/UCEC are significantly enriched in at least one GO terms, and 110/129 different enriched pathways are discovered for the identified modules. The most frequently enriched biological processes contain cell adhesion, immune response, signal transduction, cell cycle and inflammatory response. For instance, Table 2 lists the representative enriched GO terms for the selected modules in OV dataset, and we found that these modules are involved in many biological processes or pathways that related to cancers [59, 60]. For example, module 2 is enriched in *regulation of cell activation* (GO:0050865) and *immune system process* (GO:0002376), and modules 7 and 15 are enriched in *p53 signaling pathway* (KEGG: hsa04115) and *Focal adhesion* (KEGG: hsa04510), respectively. As shown in Fig. 3b and c, we also found that some enriched pathways are shared by several modules, and some of them have been reported to be involved in OV [61]. Interestingly, these two modules contain three common mRNAs (EMILIN1, COL1A2, ENC1) and one of them (COL1A2) is related to cancer, suggesting that these modules (e.g. modules 15 and 17, modules 31 and 32 in OV) with many overlaps of mRNAs are more likely to have similar biological functions.

**Table 2:** Representative enriched GO terms of the selected modules for OV dataset

Module	GO term	Description	q-value	Cancer lncRNAs	Cancer miRNAs	Cancer mRNAs
2	GO:0002376	immune system process	1.04E-12	**MALAT1**, MIR155HG, **NEAT1**, **PVT1**	mir-10a	APOC1, APOE, BTG3, C1QA, C1QB, CBS, CCL2, etc
	GO:0009605	response to external stimulus	2.31E-07			
	GO:0006954	inflammatory response	2.76E-04			
	GO:0050865	regulation of cell activation	2.25E-03			
	GO:0007154	cell communication	2.25E-03			
7	GO:0032502	developmental process	1.32E-06	DLEU2, **DNM3OS**, **GAS5**, HOTAIRM1, **MALAT1**, SNHG1, SNHG3, SNHG5, TP53TG1	mir-196b, mir-199b	CHST2,CLDN11,COX6B1, MGP, DACT3, DCHS1, DLK1, etc
	GO:0030154	cell differentiation	1.62E-05			
	GO:0060284	regulation of cell development	1.06E-04			
	GO:0010942	positive regulation of cell death	2.89E-04			
	GO:0007275	multicellular organismal development	7.77E-07			
15	GO:0007155	cell adhesion	2.57E-06	**GAS5, H19**, MEG3, SNHG5	mir-202, mir-506, mir-508, mir-513c	FSTL1, LHX1, MEST, MFAP2, CDH3, **NR5A1**, MMP2, etc
	GO:0022610	biological adhesion	2.64E-06			
	GO:0009968	negative regulation of signal transduction	1.38E-03			
	GO:0042698	ovulation cycle	3.10E-04			
	GO:0050896	response to stimulus	2.54E-05			
17	GO:0022411	cellular component disassembly	1.43E-20	**DNM3OS**, **GAS5, H19**, LINC00467, MEG3, RMRP, RP11-304 L19.5, RP11-385 J1.2, SNHG5	**mir-127**,mir-134,mir-379, **mir-370**,mir-382,mir-409, mir-410, mir-431, mir-432, **mir-433**,mir-485, mir-493, mir-654, mir-758	GPC3, **SPARC**, LHX1, LUM, MEST, MFAP2, IGF2BP2, etc
	GO:0009968	negative regulation of signal transduction	7.65E-04			
	GO:0060284	regulation of cell development	8.80E-04			
	GO:0045595	regulation of cell differentiation	5.91E-04			
	GO:0006413	translational initiation	8.31E-21			

Note: The bold letters represent the lncRNAs/miRNAs/mRNAs related to ovarian cancer; q-value represents the corrected p-value using the Benjamini-Hochberg method

Accumulating evidence has demonstrated that miRNAs located in the same cluster or belonging to the same family are likely to function synergistically or are related to the same diseases [42]. In this study, we also conducted a miRNA cluster/family enrichment analysis for the identified modules based on TAM (http://www.cuilab.cn/tam) [42]. The results indicated that 35/27 of the identified modules are significantly enriched in at least one miRNA cluster or miRNA family for OV/UCEC (*p*-value< 0.05) (Additional file 5: Table S5). For instance (see Table 3), module 1 in OV contains 9 miRNAs, 4 of which (mir-362, mir-532, mir-500, mir-501) belong to the miR-188 cluster, and three miRNAs (mir-362, mir-532, mir-501) have been supported to be associated with cancer by HMDD. Moreover, two miRNAs (mir-200b, mir-200c) in this module, which belong to the miRNA family MIPF0000019, have been shown to be related to OV [45], while another two miRNAs (mir-500, mir-501) also belong to the miRNA family MIPF0000139. As another example, two of 8 miRNAs (let-7c, mir-99a) in module 20 are from the let-7c cluster and have been shown to be dysregulated in various cancers [17]. All the findings indicate the capability of CeModule in discovering cancer-specific modules.

**Table 3:** Overlapping miRNAs for the identified modules and clusters/families in OV

Module	Overlap miRs^a^	p-value	Overlap miRs^b^	p-value
1	mir-362,mir-532, mir-500, mir-501	1.22e-06	mir-200b,mir-200c	7.33e-04
	–	–	mir-500,mir-501	2.40e-03
18	mir-99b,mir-152a	9.15e-04	mir-100,mir-99b	9.15e-04
20	let-7c, mir-99a	1.03e-04	mir-200a,mir-200b	1.01e-03
	mir-200a, mir-200b	3.07e-04	–	–
30	–	–	let-7b,let-7c	4.96e-03
70	mir-516a,mir-519a, mir-522,mir-518e	1.45e-03	mir-516a,mir-519a, mir-522,mir-518e	7.66e-04

Note: ^a/b^ represent the miRNAs that overlap between modules and miRNA clusters as well as families

### Co-expression analysis of lncRNA-miRNA-mRNA regulatory modules

We also performed an analysis to evaluate the statistical significance of (anti)-correlations between lncRNAs, miRNAs and mRNAs within modules for both datasets. We expect that the molecules within those modules identified by CeModule are more (anti)-correlated than random sets of genes. Here, we define a *correlation evaluation score* to quantify the strength of competition in any given module *C*_*v*_ as follows:

12 $$ S\left({C}_v\right)=\frac{\sum \mid {corr}_{lmiR}\mid +\sum \mid {corr}_{miRmR}\mid +\sum \mid {corr}_{lmR}\mid }{N} $$

which is defined as the average absolute values of PCCs (Pearson correlation coefficients) for all lncRNA-miRNA, miRNA-mRNA, and lncRNA-mRNA pairs, where *N* is the number of all the possible pairs for the three types of relationships in *C*_*v*_, *corr* is a function for calculating the pair-wise PCC based on the corresponding expression data.

**Fig. 4:**
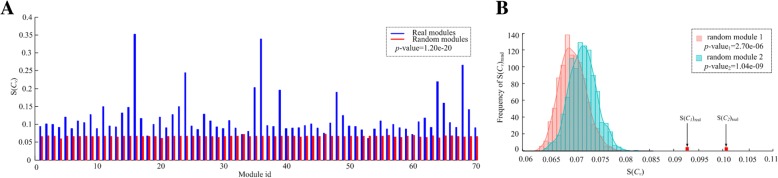
**a** Comparison of the correlation evaluation scores between all the identified modules by CeModule and the randomly generated modules for ovarian cancer dataset. **b** Distribution of the correlation evaluation scores of the 1000 random modules with the same size for modules 1 and 2 in ovarian cancer dataset

To investigate the statistical significance, we adopt a permutation test by shuffling these lncRNAs, miRNAs and mRNAs according to those identified modules, and then compute the average competing evaluation score for them. As shown in Fig. 4a, the correlation evaluation scores of our method ranged from 0.072 to 0.352 for OV, and ranged from 0.100 to 0.489 for UCEC, they exhibit significantly higher correlation than the random modules (*p*-value = 1.20e-20 for OV, p-value = 3.03e-17 for UCEC, Wilcoxon rank sum test). We can also obtain the same conclusions on the two examples for modules 1 (p-value = 2.70e-06, Student’s t-test) and 2 (p-value = 1.04e-09) (Fig. 4b). Here, the correlation evaluation scores of these identified modules are generally weak, this is mainly due to the fact that the vast majority of Pearson correlation coefficients (PCCs) of lncRNA-miRNA, miRNA-mRNA and lncRNA-mRNA pairs were weak in the used datasets of OV and UCEC (Table 4).

**Table 4:** Statistics of the correlation coefficients in OV and UCEC datasets

Dataset	Ave (lnc-miR)	Ave (miR-mR)	Ave (lnc-mR)	Ave-mod
OV	0.0546	0.0659	0.0678	0.119
UCEC	0.0639	0.0772	0.0854	0.173

Note: Ave (lnc-miR), Ave (miR-mR) and Ave (lnc-mR) are the average absolute Pearson correlation coefficients of all lncRNA-miRNA, miRNA-mRNA and lncRNA-mRNA pairs, respectively; Ave-mod is the correlation evaluation score across all modules

### Regulatory modules are strongly implicated in cancer

Base on the fact that the input data included the lncRNA, miRNA and mRNA expression profiles of OV and UCEC samples, we expect the modules indentified by our method to be related to cancers, especially OV/UCEC. Here, we obtained 82/265/4288 (116/322/4721) cancer-related lncRNAs/miRNAs/mRNAs that are involved in the expression profiles as the benchmark sets for OV (UCEC), and collected 11/5 lncRNAs, 83/75 miRNAs and 73/158 mRNAs related to OV/UCEC from several reliable databases as mentioned in the Section of Methods.

**Fig. 5:**
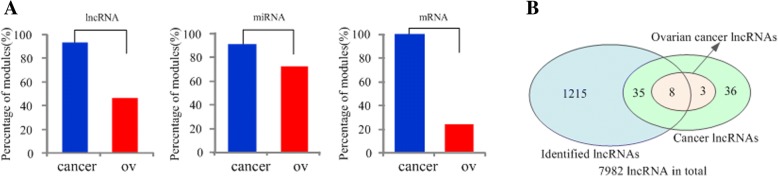
**a** Percentage of modules with at least two known cancer-related (ovarian cancer-related)lncRNAs/miRNAs/mRNAs in ovarian cancer dataset. **b** Overlap of cancer lncRNAs, and ovarian cancer lncRNAs between the benchmark set and lncRNAs in the identified modules for ovarian cancer dataset

As shown in Fig. 5a, 45.7% (92.9%), 71.4% (90.0%) and 22.9% (100%) of all the identified modules in OV dataset contained at least two OV-related (cancer-related) lncRNAs, miRNAs and mRNAs, respectively. Meanwhile, the corresponding ratios in UCEC dataset are 1.4% (62.9%), 64.3% (91.4%) and 10.0% (100%) for uterine corpus endometrial carcinoma-related (cancer-related) lncRNAs, miRNAs and mRNAs. The significant level of overlap between every module and cancer (OV/UCEC) lncRNAs/miRNAs/mRNAs is evaluated by hypergeometric test, and Table 5 lists the OV-related and cancer-related lncRNAs for several representative modules. For example, module 66 in OV dataset contains 58 lncRNAs, 9 of which are cancer lncRNAs and 6 of them are ovarian cancer lncRNAs. To take another example, module 51 in UCEC dataset contains 61 lncRNAs, 8 of which are cancer lncRNAs and 3 of them are uterine corpus endometrial carcinoma-related lncRNAs. We provided all the cancer (OV/UCEC) related modules for both datasets in Additional file 6: Table S6.

**Table 5:** Known ovarian cancer-associated and cancer- associated lncRNAs for these representative modules in OV

Module	Cancer lncRNAs	Num^a^	q-value	OV lncRNAs	Num^b^	q-value
2	MALAT1,MIR155HG,NEAT1,PVT1	4/74	1.05e-02	MALAT1, NEAT1, PVT1	3/74	4.65e-04
7	DLEU2,DNM3OS,GAS5,HOTAIRM1,MALAT1, SNHG1,SNHG3,SNHG5,TP53TG1	9/86	2.30e-06	DNM3OS, GAS5, MALAT1	3/86	6.55e-04
12	MALAT1,RMRP,RP11-385 J1.2,XIST	4/30	6.00e-04	MALAT1, XIST	2/30	2.34e-03
31	GAS5,MALAT1,NEAT1,RP11-304 L19.5,SNHG3,SNHG5,TP53TG1,UCA1	8/75	6.59e-06	GAS5,MALAT1,NEAT1, UCA1	4/75	3.95e-05
41	DLEU2,GAS5,LINC00467,MALAT1,NEAT1, SNHG1,SNHG3	7/57	9.50e-06	GAS5, MALAT1, NEAT1	3/57	3.91e-04
62	DNM3OS,H19,HOTAIRM1,LINC00152,MALAT1,MEG3,NEAT1,PVT1,RMRP,RP11-304 L19.5,RP11-401P9.4,SNHG5,UCA1,XIST	14/79	2.35e-12	DNM3OS, MALAT1, PVT1, NEAT1, UCA1, XIST, H19	7/79	1.59e-10
66	H19,MALAT1,MEG3,NEAT1,PVT1,SNHG1, SNHG3,UCA1,XIST	9/58	2.02e-07	H19, MALAT1, NEAT1, PVT1, UCA1, XIST	6/58	1.78e-09

Note: Num^a^ and Num^b^ are the ratios of lncRNAs that associated with cancer and OV in these modules. q-value is the FDR-corrected p-value after multiple testing correction

For OV (UCEC) dataset, the identified modules involve 1258/171/2172 (1252/172/2498) different lncRNAs/miRNAs/mRNAs. In the results of OV, as shown in Fig. 5b, 43 lncRNAs belong to the benchmark set of cancer lncRNAs (*p*-value = 1.18e-14, hypergeometric test), and 8 of them are relevant to ovarian cancer (*p*-value = 3.93e-05). In UCEC, 47 lncRNAs in those modules belong to the corresponding benchmark set (*p*-value = 6.05e-11) and 3 of which are UCEC specific lncRNAs (*p*-value = 2.93e-02). For miRNAs, 64.9%/77.3% of the 171/172 miRNAs are known to be involved in cancer in both datasets, and 51/43 miRNAs are specifically associated with OV/UCEC (*p*-value = 2.70e-05 for OV, *p*-value = 6.29e-06 for UCEC). Meanwhile, 1058/1186 mRNAs have been verified to be related to cancer, and 27/29 mRNAs are confirmed to be associated with ovarian cancer and uterine corpus endometrial carcinoma in OV and UCEC datasets, respectively. All the cancer-related and OV (UCEC) related molecules in those modules for both datasets are listed in Additional file 6: Table S6.

We also performed a differential expression analysis by two-sample t-test for those OV-related miRNAs (83 miRNAs) to investigate the cancer-specific abnormal changes in expression profile data. As a result, we identified 13 differentially expressed miRNAs (mir-200c, mir-99b, mir-183, mir-187, mir-10b, mir-625, mir-92b, mir-182, mir-449b, mir-107, mir-134, mir-98, mir-141, Additional file 7: Table S7) from those miRNAs, and found that 62.9% (44/70, Additional file 7: Table S7) of the modules contain at least one miRNAs that are differential expression. There are four modules (modules 13, 57, 60, and 69) are significantly enriched in ovarian cancer related differentially expressed miRNAs (hypergeometric test, FDR < 0.05, Additional file 7: Table S7). For example, module 57 contains 5 OV-related miRNAs (mir-182, mir-183, mir-200c, mir-625, mir-99b) and all of them are differential expression (FDR = 2.40e-05). The above observations imply that the lncRNAs/miRNAs/mRNAs in the identified modules are involved in various cancers, which confirm that the proposed method has a potential capability to discover modules related to cancers.

## Discussion

Increasing evidence indicates that a novel competitive endogenous RNA (ceRNA) regulatory mechanism exists between non-coding RNAs and protein-coding RNAs. LncRNAs and miRNAs are two kinds of crucial regulators and participate in many important biological processes. The aberrant expression of lncRNAs and miRNAs often contribute to tumorigenesis. To utilize the tremendous amounts of heterogeneous omics data and investigate the synergistic and cooperative mechanisms involve in lncRNAs, miRNAs, and mRNAs, our method integrates lncRNA/miRNA/mRNA expression profile data in an NMF framework, and simultaneously incorporates interaction networks in a regularized manner. The results of both (OV/UCEC) datasets indicate that the modules identified by CeModule contain many lncRNAs/miRNAs/mRNAs with specific topological patterns that are involved in some crucial biological processes and may cause cancers. Meanwhile, we further investigated whether the discovered modules were associated with the survival of ovarian cancer patients. The clinical data are downloaded from TCGA, and 383 samples are retained after removing those not included in the expression data or those with unavailable survival time. Kaplan-Meier survival analysis also indicates the ability of the method to discover modules that provide useful information for the prediction of cancer prognosis (Additional file 1).

## Conclusions

In this study, we systematically investigate the efficiency of CeModule in identifying biologically functional modules that related to specific biological processes or cancers. We applied our method on the lncRNA/miRNA/mRNA expression data with matched samples of ovarian cancer and uterine corpus endometrial carcinoma from TCGA, and finally obtained 70 regulatory modules in both datasets. The observations indicate that these modules are densely connected and show specific topological characteristics. Meanwhile, these modules are significantly associated with many disease-related biological processes and pathways. Furthermore, a large number of lncRNAs/miRNAs/mRNAs in the modules are involved in various human complex diseases, such as ovarian cancer. All the results fully demonstrate the capability of CeModule for identifying of biologically functional modules. As a large number of sample-matched lncRNAs/miRNAs/mRNAs expression profile data become available, we believe that CeModule can serve as a potential tool for revealing condition-specific ceRNA regulatory patterns for cancer.

## Additional files

Additional file 1: **Figure S1.** Topological features of the identified modules and the ceRNA regulatory module network. The distributions of number of **(A)** lncRNAs, **(B)** miRNAs, and **(C)** mRNAs for the identified modules in OV dataset. **Figure S2.** Topological features of the identified modules and the ceRNA regulatory module network. The distributions of number of **(A)** lncRNAs, **(B)** miRNAs, and **(C)** mRNAs for the identified modules in UCEC dataset. **Figure S3.** Overlap of the top 10 (A) miRNAs and (B) mRNAs across three dimensions (degree, betweenness centrality, and closeness centrality) in OV dataset. **Figure S4.** Overlap of the top 10 (A) lncRNAs, (B) miRNAs and (C) mRNAs across three dimensions (degree, betweenness centrality, and closeness centrality) in UCEC dataset. **Figure S5** Kaplan-Meier survival curves for ovarian cancer patients classified into two groups using the module-averaged lncRNA expression levels. **Table S2**. The top 10 lncRNAs, miRNAs and mRNAs with the highest degree, closeness centrality, and betweenness centrality in UCEC. (PDF 525 kb)

Additional file 2: **Table S1.** The list of all the identified modules that involving lncRNAs, miRNAs and mRNAs. (XLSX 248 kb)

Additional file 3: **Table S3.** Results of the enriched GO biological processes for the identified modules. (XLSX 620 kb)

Additional file 4: **Table S4.** Results of the enriched KEGG pathways for the identified modules. (XLSX 105 kb)

Additional file 5: **Table S5.** The list of regulatory modules enriched in miRNA cluster and miRNA family. (XLSX 18 kb)

Additional file 6: **Table S6.** Known OV/UCEC-related lncRNAs/miRNAs/mRNAs and cancer-related lncRNAs/miRNAs/mRNAs in modules. (XLSX 52 kb)

Additional file 7: **Table S7.** Differentially expressed miRNAs identified in modules. (XLSX 14 kb)

## Abbreviations

ceRNA: Competing endogenous RNA
EA: Electronic Annotation
GO: Gene Ontology
lncRNAs: Long non-coding RNAs
MREs: miRNA response elements
NAS: Nontraceable Author Statement
ncRNAs: Non-coding RNAs
ND: No biological Data available
NMF: Non-negative Matrix Factorization
OV: Ovarian Cancer
TCGA: The Cancer Genome Atlas

## References

[1] Jopling CL, Yi MK, Lancaster AM, Lemon SM, Sarnow P (2005). Modulation of hepatitis C virus RNA abundance by a liver-specific microRNA. Science.

[2] Yang Z, Wu L, Wang A, Tang W, Zhao Y, Zhao H, Teschendorff AE (2017). dbDEMC 2.0: updated database of differentially expressed miRNAs in human cancers. Nucleic Acids Res.

[3] Jin D, Lee H (2015). A computational approach to identifying gene-microRNA modules in cancer. PLoS Comput Biol.

[4] Karp X, Ambros V (2005). Developmental biology. Encountering microRNAs in cell fate signaling. Science.

[5] Cheng AM, Byrom MW, Shelton J, Ford LP (2005). Antisense inhibition of human miRNAs and indications for an involvement of miRNA in cell growth and apoptosis. Nucleic Acids Res.

[6] Xiao Q, Luo J, Liang C, Li G, Cai J, Ding P, Liu Y (2018). Identifying lncRNA and mRNA co-expression modules from matched expression data in ovarian Cancer. IEEE/ACM Trans Comput Biol Bioinform.

[7] Zeng XX, Zhang X, Zou Q (2016). Integrative approaches for predicting microRNA function and prioritizing disease-related microRNA using biological interaction networks. Brief Bioinform.

[8] Luo J, Xiao Q (2017). A novel approach for predicting microRNA-disease associations by unbalanced bi-random walk on heterogeneous network. J Biomed Inform.

[9] Salmena L, Poliseno L, Tay Y, Kats L, Pandolfi PP (2011). A ceRNA hypothesis: the Rosetta stone of a hidden RNA language?. Cell.

[10] Poliseno L, Salmena L, Zhang JW, Carver B, Haveman WJ, Pandolfi PP (2010). A coding-independent function of gene and pseudogene mRNAs regulates tumour biology. Nature.

[11] Ebert MS, Neilson JR, Sharp PA (2007). MicroRNA sponges: competitive inhibitors of small RNAs in mammalian cells. Nat Methods.

[12] Wang JY, Liu XF, Wu HC, Ni PH, Gu ZD, Qiao YX, Chen N, Sun FY, Fan QS (2010). CREB up-regulates long non-coding RNA, HULC expression through interaction with microRNA-372 in liver cancer. Nucleic Acids Res.

[13] Franco-Zorrilla JM, Valli A, Todesco M, Mateos I, Puga MI, Rubio-Somoza I, Leyva A, Weigel D, Garcia JA, Paz-Ares J (2007). Target mimicry provides a new mechanism for regulation of microRNA activity. Nat Genet.

[14] Chiu YC, Wang LJ, Lu TP, Hsiao TH, Chuang EY, Chen Y. Differential correlation analysis of glioblastoma reveals immune ceRNA interactions predictive of patient survival. BMC Bioinformatics. 2017:18(1):132. 10.1186/s12859-017-1557-4 PMC5330036 28241741

[15] Tay Y, Rinn J, Pandolfi PP (2014). The multilayered complexity of ceRNA crosstalk and competition. Nature.

[16] Liang C, Li Y, Luo JW, Zhang ZL (2015). A novel motif-discovery algorithm to identify co-regulatory motifs in large transcription factor and microRNA co-regulatory networks in human. Bioinformatics.

[17] Liang C, Li Y, Luo J (2016). A novel method to detect functional microRNA regulatory modules by Bicliques merging. IEEE/ACM Trans Comput Biol Bioinform.

[18] Li Y, Liang C, Wong KC, Luo J, Zhang Z (2014). Mirsynergy: detecting synergistic miRNA regulatory modules by overlapping neighbourhood expansion. Bioinformatics.

[19] Zhang J, Le TD, Liu L, He J, Li J (2016). A novel framework for inferring condition-specific TF and miRNA co-regulation of protein-protein interactions. Gene.

[20] Xiao Q, Luo JW, Liang C, Cai J, Ding PJ (2018). A graph regularized non-negative matrix factorization method for identifying microRNA-disease associations. Bioinformatics.

[21] Luo JW, Xiao Q, Liang C, Ding PJ (2017). Predicting MicroRNA-disease associations using Kronecker regularized least squares based on heterogeneous omics data. IEEE Access.

[22] Xu J, Li Y, Lu J, et al. The mRNA related ceRNA-ceRNA landscape and significance across 20 major cancer types. Nucleic Acids Res. 2015;43:8169–82. 10.1093/nar/gkv853 PMC4787795 26304537

[23] Song C, Zhang J, Liu Y, Pan H, Qi HP, Cao YG, Zhao JM, Li S, Guo J, Sun HL (2016). Construction and analysis of cardiac hypertrophy-associated lncRNA-mRNA network based on competitive endogenous RNA reveal functional lncRNAs in cardiac hypertrophy. Oncotarget.

[24] Feng L, Wang R, Lian M, Ma H, He N, Liu H, Wang H, Fang J (2016). Integrated analysis of long noncoding RNA and mRNA expression profile in advanced laryngeal squamous cell carcinoma. PLoS One.

[25] Xia T, Liao Q, Jiang X, Shao Y, Xiao B, Xi Y, Guo J (2014). Long noncoding RNA associated-competing endogenous RNAs in gastric cancer. Sci Rep.

[26] Paraskevopoulou MD, Georgakilas G, Kostoulas N, Reczko M, Maragkakis M, Dalamagas TM, Hatzigeorgiou AG (2013). DIANA-LncBase: experimentally verified and computationally predicted microRNA targets on long non-coding RNAs. Nucleic Acids Res.

[27] Liu K, Yan Z, Li Y, Sun Z (2013). Linc2GO: a human LincRNA function annotation resource based on ceRNA hypothesis. Bioinformatics.

[28] Chen G, Wang ZY, Wang DQ, Qiu CX, Liu MX, Chen X, Zhang QP, Yan GY, Cui QH (2013). LncRNADisease: a database for long-non-coding RNA-associated diseases. Nucleic Acids Res.

[29] Wang JJY, Wang XL, Gao X (2013). Non-negative matrix factorization by maximizing correntropy for cancer clustering. BMC Bioinformatics.

[30] Ievgen R, Younes B (2014). Controlling orthogonality constraints for better NMF clustering. International Joint Conference on Neural Networks.

[31] Ding C, Li T, Peng W, Park H (2006). Orthogonal nonnegative matrix t-factorizations for clustering. Proceedings of the 12th ACM SIGKDD international conference on knowledge discovery and data mining.

[32] Chin L, Meyerson M, Aldape K, Bigner D, Mikkelsen T, VandenBerg S, Kahn A, Penny R, Ferguson ML, Gerhard DS (2008). Comprehensive genomic characterization defines human glioblastoma genes and core pathways. Nature.

[33] Liu X, Wang WJ, He DX, Jiao PG, Jin D, Cannistraci CV (2017). Semi-supervised community detection based on non-negative matrix factorization with node popularity. Inform Sci.

[34] Yoo J, Choi S (2010). Nonnegative matrix factorization with orthogonality constraints. Manag Sci.

[35] Wang YX, Zhang YJ (2013). Nonnegative matrix factorization: a comprehensive review. Ieee T Knowl Data En.

[36] Hoyer PO (2004). Non-negative matrix factorization with sparseness constraints. J Mach Learn Res.

[37] Lee DD, Seung HS (1999). Learning the parts of objects by non-negative matrix factorization. Nature.

[38] Facchinei F, Kanzow C, Sagratella S (2014). Solving quasi-variational inequalities via their KKT conditions. Math Program.

[39] Xiao Q, Luo JW, Dai JH. Computational prediction of human disease-associated circRNAs based on manifold regularization Learning framework. IEEE J Biomed Health Inform. 2019. 10.1109/JBHI.2019.2891779. 10.1109/JBHI.2019.2891779 30629521

[40] Chen JY, Zhang SH (2016). Integrative analysis for identifying joint modular patterns of gene-expression and drug-response data. Bioinformatics.

[41] Subramanian A, Tamayo P, Mootha VK, Mukherjee S, Ebert BL, Gillette MA, Paulovich A, Pomeroy SL, Golub TR, Lander ES (2005). Gene set enrichment analysis: a knowledge-based approach for interpreting genome-wide expression profiles. P Natl Acad Sci USA.

[42] Lu M, Shi B, Wang JA, Cao Q, Cui QH (2010). TAM: a method for enrichment and depletion analysis of a microRNA category in a list of microRNAs. BMC Bioinformatics.

[43] Kozomara A, Griffiths-Jones S (2014). miRBase: annotating high confidence microRNAs using deep sequencing data. Nucleic Acids Res.

[44] Ning SW, Zhang JZ, Wang P, Zhi H, Wang JJ, Liu Y, Gao Y, Guo MN, Yue M, Wang LH (2016). Lnc2Cancer: a manually curated database of experimentally supported lncRNAs associated with various human cancers. Nucleic Acids Res.

[45] Li Y, Qiu CX, Tu J, Geng B, Yang JC, Jiang TZ, Cui QH (2014). HMDD v2.0: a database for experimentally supported human microRNA and disease associations. Nucleic Acids Res.

[46] Pinero J, Bravo A, Queralt-Rosinach N, Gutierrez-Sacristan A, Deu-Pons J, Centeno E, Garcia-Garcia J, Sanz F, Furlong LI (2017). DisGeNET: a comprehensive platform integrating information on human disease-associated genes and variants. Nucleic Acids Res.

[47] Akrami R, Jacobsen A, Hoell J, Schultz N, Sander C, Larsson E. Comprehensive Analysis of Long Non-Coding RNAs in Ovarian Cancer Reveals Global Patterns and Targeted DNA Amplification. Plos One. 2013;8(11):e80306. 10.1371/journal.pone.0080306 PMC3827191 24265805

[48] Min W, Liu J, Luo F, Zhang S (2016). A two-stage method to identify joint modules from matched MicroRNA and mRNA expression data. Ieee T Nanobiosci.

[49] Kim H, Park H (2007). Sparse non-negative matrix factorizations via alternating non-negativity-constrained least squares for microarray data analysis. Bioinformatics.

[50] Li JH, Liu S, Zhou H, Qu LH, Yang JH (2014). starBase v2.0: decoding miRNA-ceRNA, miRNA-ncRNA and protein-RNA interaction networks from large-scale CLIP-Seq data. Nucleic Acids Res.

[51] Xiao FF, Zuo ZX, Cai GS, Kang SL, Gao XL, Li TB (2009). miRecords: an integrated resource for microRNA-target interactions. Nucleic Acids Res.

[52] Vergoulis T, Vlachos IS, Alexiou P, Georgakilas G, Maragkakis M, Reczko M, Gerangelos S, Koziris N, Dalamagas T, Hatzigeorgiou AG (2012). TarBase 6.0: capturing the exponential growth of miRNA targets with experimental support. Nucleic Acids Res.

[53] Hsu SD, Tseng YT, Shrestha S, Lin YL, Khaleel A, Chou CH, Chu CF, Huang HY, Lin CM, Ho SY (2014). miRTarBase update 2014: an information resource for experimentally validated miRNA-target interactions. Nucleic Acids Res.

[54] Lee I, Blom UM, Wang PI, Shim JE, Marcotte EM (2011). Prioritizing candidate disease genes by network-based boosting of genome-wide association data. Genome Res.

[55] Li GB, Zhang HH, Wan XS, Yang XB, Zhu CP, Wang AQ, He L, Miao RY, Chen SG, Zhao HT. Long noncoding RNA plays a key role in metastasis and prognosis of hepatocellular carcinoma. BioMed Research International. 2014;2014:1–8. 10.1155/2014/780521 PMC3976793 24757675

[56] Gutschner T, Hammerle M, Eissmann M, Hsu J, Kim Y, Hung G, Revenko A, Arun G, Stentrup M, Gross M (2013). The noncoding RNA MALAT1 is a critical regulator of the metastasis phenotype of lung Cancer cells. Cancer Res.

[57] Sun M, Xia R, Jin FY, Xu TP, Liu ZJ, De W, Liu XH (2014). Downregulated long noncoding RNA MEG3 is associated with poor prognosis and promotes cell proliferation in gastric cancer. Tumor Biol.

[58] Ying L, Huang YR, Chen HG, Wang YW, Xia L, Chen YH, Liu YD, Qiu F (2013). Downregulated MEG3 activates autophagy and increases cell proliferation in bladder cancer. Mol BioSyst.

[59] Kupryjanczyk J, Thor AD, Beauchamp R, Merritt V, Edgerton SM, Bell DA, Yandell DW (1993). p53 gene mutations and protein accumulation in human ovarian cancer. Proc Natl Acad Sci U S A.

[60] Li Z, Gou J, Xu J (2013). Down-regulation of focal adhesion signaling in response to cyclophilin a knockdown in human endometrial cancer cells, implicated by cDNA microarray analysis. Gynecol Oncol.

[61] Kwon Y, Cukierman E, Godwin AK (2011). Differential expressions of adhesive molecules and proteases define mechanisms of ovarian tumor cell matrix penetration/invasion. PLoS One.

