# Supplementary material for: CeModule: an integrative framework for discovering regulatory patterns from genomic data in cancer
Source: BMC Bioinformatics. 2019 Feb 7;20:67. doi: 10.1186/s12859-019-2654-3 (PMC6367773; doi:10.1186/s12859-019-2654-3)
Supplement: Supplementary file 1 — Figure S1. Topological features of the identified modules and the ceRNA regulatory module network. The distributions of number of (A) lncRNAs, (B) miRNAs, and (C) mRNAs for the identified modules in OV dataset. Figure S2. Topological features of the identified modules and the ceRNA regulatory module network. The distributions of number of (A) lncRNAs, (B) miRNAs, and (C) mRNAs for the identified modules in UCEC dataset. Figure S3. Overlap of the top 10 (A) miRNAs and (B) mRNAs across three dimensions (degree, betweenness centrality, and closeness centrality) in OV dataset. Figure S4. Overlap of the top 10 (A) lncRNAs, (B) miRNAs and (C) mRNAs across three dimensions (degree, betweenness centrality, and closeness centrality) in UCEC dataset. Figure S5 Kaplan-Meier survival curves for ovarian cancer patients classified into two groups using the module-averaged lncRNA expression levels. Table S2. The top 10 lncRNAs, miRNAs and mRNAs with the highest degree, closeness centrality, and betweenness centrality in UCEC. (PDF 525 kb) [file 12859_2019_2654_MOESM1_ESM.pdf]

CeModule: an integrative framework for discovering regulatory patterns from genomic data in cancer

Qiu Xiao[1,2], Jiawei Luo[1,*], Cheng Liang[3], Jie Cai[1], Guanghui Li[1] and Buwen Cao[1]

[1]College of Computer Science and Electronic Engineering, Hunan University, Changsha, 410083, China, [2]College of Information Science and Engineering, Hunan Normal University, Changsha 410081, China and [3]College of Information Science and Engineering, Shandong Normal University, Jinan, 250000, China

Supplementary Materials

S1. Derivation of the CeModule algorithm

The optimization problem of CeModule can be mathematically formulated as follows:

$$\begin{aligned}\min_{W,H_1,H_2,H_3} &\sum_{i=1,2,3}(\| X_i - WH_i^T \|_F^2 + \frac{1}{2}\alpha \| H_i^T H_i - I \|_F^2) \\ &-\lambda_1 Tr(H_2^T AH_1) - \lambda_2 Tr(H_2^T BH_3) - \lambda_3 Tr(H_3^T CH_3) \\ &+\gamma_1 \| W \|_F^2 + \gamma_2 \sum_{i=1,2,3} \| H_i \|_1 \\ &s.t. \quad W \geq 0, H_i \geq 0\end{aligned} \quad (1)$$

For the Standard NMF problem, although the objective function of NMF is convex in W only or H only, it is not convex in both variables together. Obviously, the above objective function is not convex on W, H_1, H_2 and H_3, and it is unreasonable to find its global minimum. In the following, as the same way for the original NMF, we adopt an iterative update algorithm to find local minimum of the problem by updating W and H_i iteratively. Below we detail the multiplicative updating algorithm for CeModule to identify the local minimum of the objective function F:

$$\begin{aligned}F = &\sum_{i=1,2,3}(\| X_i - WH_i^T \|_F^2 + \frac{1}{2}\alpha \| H_i^T H_i - I \|_F^2) \\ &-\lambda_1 Tr(H_2^T AH_1) - \lambda_2 Tr(H_2^T BH_3) - \lambda_3 Tr(H_3^T CH_3) \\ &+\gamma_1 \| W \|_F^2 + \gamma_2 \sum_{i=1,2,3} \| H_i \|_1\end{aligned} \quad (2)$$

Based on the simple knowledge of linear algebra, the objective function F can be rewritten as follows:

$$\begin{aligned}F = &\sum_{i=1}^{3}\Big[Tr(X_i X_i^T) - 2Tr(X_i H_i W^T) + Tr(WH_i^T H_i W^T) \\ &+\frac{1}{2}\alpha\Big(Tr(H_i^T H_i H_i^T H_i) - 2Tr(H_i^T H_i) + Tr(I^T I)\Big)\Big] \\ &-\lambda_1 Tr(H_2^T AH_1) - \lambda_2 Tr(H_2^T BH_3) - \lambda_3 Tr(H_3^T CH_3) \\ &+\gamma_1 Tr(WW^T) + \gamma_2 \sum_{i=1}^{3} Tr(E_i^T H_i)\end{aligned} \quad (3)$$

where $E_1 \in \{1\}^{N1\times K}$, $E_2 \in \{1\}^{N2\times K}$, and $E_3 \in \{1\}^{N3\times K}$. Let $\Phi=[\varphi_{lk}]$, $\Psi=[\psi_{jk}]$, $\Omega=[\omega_{pk}]$, and $\Theta=[\theta_{qk}]$ be the Lagrange multipliers for the constrains $w_{lk}\geq 0$, $h_{jk}^{(1)}\geq 0$, $h_{pk}^{(2)}\geq 0$, and $h_{pk}^{(3)}\geq 0$, respectively. The corresponding Lagrange function L_f of Eq. (3) is defined as:

$$L_f = F + Tr(\Phi W^T) + Tr(\Psi H_1^T) + Tr(\Omega H_2^T) + Tr(\Theta H_3^T) \quad (4)$$

The partial derivatives of the above function with respect to W and H_i are:

$$\begin{aligned}\frac{\partial L_f}{\partial W} &= \sum_{i=1}^{3}\Big[-2X_i H_i + 2WH_i^T H_i\Big] + 2\gamma_1 W + \Phi \\ \frac{\partial L_f}{\partial H_1} &= -2X_1^T W + 2H_1 W^T W + \tfrac{1}{2}\alpha(4H_1 H_1^T H_1 - 4H_1) \\ &\quad -\lambda_1 A^T H_2 + \gamma_2 E_1 + \Psi \\ \frac{\partial L_f}{\partial H_2} &= -2X_2^T W + 2H_2 W^T W + \tfrac{1}{2}\alpha(4H_2 H_2^T H_2 - 4H_2) \\ &\quad -\lambda_1 AH_1 - \lambda_2 BH_3 + \gamma_2 E_2 + \Omega \\ \frac{\partial L_f}{\partial H_3} &= -2X_3^T W + 2H_3 W^T W + \tfrac{1}{2}\alpha(4H_3 H_3^T H_3 - 4H_3) \\ &\quad -\lambda_2 B^T H_2 - 2\lambda_3 CH_3 + \gamma_2 E_3 + \Theta\end{aligned} \quad (5)$$

Using the Karush-Kuhn-Tucker (KKT) conditions $\varphi_{lk}w_{lk}=0$, $\psi_{jk}h_{jk}^{(1)}=0$, $\omega_{pk}h_{pk}^{(2)}=0$, and $\theta_{qk}h_{pk}^{(3)}=0$, we obtain the following equations for w_{lk}, $h_{jk}^{(1)}$, $h_{pk}^{(2)}$, and $h_{pk}^{(3)}$:

$$\begin{aligned}&-2\sum_{i=1}^{3}(X_i H_i)_{lk} w_{lk} + 2\Big[\sum_{i=1}^{3}(WH_i^T H_i) + (\gamma_1 W)\Big]_{lk} w_{lk} = 0 \\ &(-2X_1^T W - 2\alpha H_1 - \lambda_1 A^T H_2)_{jk} h_{jk}^{(1)} \\ &\qquad + (2H_1 W^T W + 2\alpha H_1 H_1^T H_1 + \gamma_2 E_1)_{jk} h_{jk}^{(1)} = 0 \\ &(-2X_2^T W - 2\alpha H_2 - \lambda_1 AH_1 - \lambda_2 BH_3)_{pk} h_{pk}^{(2)} \\ &\qquad + (2H_2 W^T W + 2\alpha H_2 H_2^T H_2 + \gamma_2 E_2)_{pk} h_{pk}^{(2)} = 0 \\ &(-2X_3^T W - 2\alpha H_3 - \lambda_2 B^T H_2 - 2\lambda_3 CH_3)_{qk} h_{qk}^{(3)} \\ &\qquad + (2H_3 W^T W + 2\alpha H_3 H_3^T H_3 + \gamma_2 E_3)_{qk} h_{qk}^{(3)} = 0\end{aligned} \quad (6)$$

Then, we determine the multiplicative update rules for W and H_i as follows:

$$\begin{aligned}w_{lk} &\leftarrow w_{lk}\frac{(X_1 H_1 + X_2 H_2 + X_3 H_3)_{lk}}{(WH_1^T H_1 + WH_2^T H_2 + WH_3^T H_3 + \gamma_1 W)_{lk}} \\ h_{jk}^{(1)} &\leftarrow h_{jk}^{(1)}\frac{(X_1^T W + \alpha H_1 + \frac{\lambda_1}{2}A^T H_2)_{jk}}{(H_1 W^T W + \alpha H_1 H_1^T H_1 + \frac{\gamma_2}{2}E_1)_{jk}} \\ h_{pk}^{(2)} &\leftarrow h_{pk}^{(2)}\frac{(X_2^T W + \alpha H_2 + \frac{\lambda_1}{2}AH_1 + \frac{\lambda_2}{2}BH_3)_{pk}}{(H_2 W^T W + \alpha H_2 H_2^T H_2 + \frac{\gamma_2}{2}E_2)_{pk}} \\ h_{qk}^{(3)} &\leftarrow h_{qk}^{(3)}\frac{(X_3^T W + \alpha H_3 + \frac{\lambda_2}{2}B^T H_2 + \lambda_3 CH_3)_{qk}}{(H_3 W^T W + \alpha H_3 H_3^T H_3 + \frac{\gamma_2}{2}E_3)_{qk}}\end{aligned} \quad (7)$$

S2. Convergence analysis of the CeModule algorithm

We have the following theorem to guarantee the convergence of the above updating rules to a local optimum.

Theorem 1 The objective function F of the CeModule problem is non-increasing under the above updating rules in Eq. (7). The objective function is finite and invariant under these updates if and only if W, H_1, H_2, and H_3 are at a stationary point.

The principle of convergence proof of NMF can be easily expanded to prove this theorem. A difference to NMF is that the objective function F here can be unbounded below. Only the objective is finite, the CeModule can get a stable local solution. Here, we show that F is non-increasing under the updating rules. In particular, here we prove that the F is non-increasing under the updating rule for W, and same feature under the updating rules for H_1, H_2, and H_3 can be similarly proved. We will adopt the same strategy used in (Lee and Seung, 2001) that introduced an auxiliary function in the Expectation-Maximization algorithm. The following is the definition of the auxiliary function.

Definition $G(w,w')$ is an auxiliary function of $F(w)$ if the following conditions are satisfied:

$$\begin{aligned} &G(w,w') \ge F(w) \qquad \forall w, \\ &G(w,w) = F(w). \end{aligned} \tag{8}$$

The above auxiliary function is very important because of the following lemma.

Lemma 1: *If G is an auxiliary function for F, then F is non-increasing under the update*

$$w^{(t+1)} = \arg\min_{w} G(w, w^{(t)}) \tag{9}$$

Proof:

$$F(w^{(t+1)}) \le G(w^{(t+1)}, w^{(t)}) \le G(w^{(t)}, w^{(t)}) = F(w^{(t)}).$$

Now, we show that the updating rule for W is exactly the update in this Lemma with a proper auxiliary function.

Considering any element $(W)_{ab}$ in W, we use the F_{ab} to denote the part of F, which is only relevant to $(W)_{ab}$. It is easy to check that

$$F'_{ab} = \left(\frac{\partial F}{\partial W}\right)_{ab} = \left(\sum_{i=1}^{3}\left[-2X_iH_i + 2WH_i^TH_i\right] + 2\gamma_1 W\right)_{ab} \tag{10}$$

$$F''_{ab} = \left(\frac{\partial^2 F}{\partial W^2}\right)_{ab} = 2\left(\sum_{i=1}^{3}\left(H_i^TH_i\right) + \lambda_1 I\right)_{bb} \tag{11}$$

Lemma 2: *Function*

$$\begin{aligned} G(w, w_{ab}^{(t)}) &= F_{ab}(w_{ab}^{(t)}) + F'_{ab}(w_{ab}^{(t)})(w - w_{ab}^{(t)}) \\ &+ \frac{\left(W\sum_{i=1}^{3}\left(H_i^TH_i\right) + \lambda_1 W\right)_{ab}}{w_{ab}^{(t)}}(w - w_{ab}^{(t)})^2 \end{aligned} \tag{12}$$

is an auxiliary function for F_{ab}.

Proof: Since $G(w, w) = F_{ab}(w)$ is obvious, we only need show that $G(w, w_{ab}^{(t)}) \ge F_{ab}(w)$. To do this, we first consider the Taylor series expansion of $F_{ab}(w)$

$$\begin{aligned} F_{ab}(w) &= F_{ab}(w_{ab}^{(t)}) + F'_{ab}(w_{ab}^{(t)})(w - w_{ab}^{(t)}) \\ &+ \left(\sum_{i=1}^{3}\left(H_i^TH_i\right) + \lambda_1 I\right)_{bb}(w - w_{ab}^{(t)})^2 \end{aligned} \tag{13}$$

We compare Eq.(12) with Eq.(13) to find that $G(w, w_{ab}^{(t)}) \ge F_{ab}(w)$ is equivalent to

$$\begin{aligned} \frac{\left(W\sum_{i=1}^{3}\left(H_i^TH_i\right) + \lambda_1 W\right)_{ab}}{w_{ab}^{(t)}} &\ge \frac{F''_{ab}(w_{ab}^{(t)})}{2} \\ &= \left(\sum_{i=1}^{3}\left(H_i^TH_i\right) + \lambda_1 I\right)_{bb} \end{aligned} \tag{14}$$

Obviously, we have

$$\begin{aligned} \left(W\sum_{i=1}^{3}\left(H_i^TH_i\right) + \lambda_1 W\right)_{ab} &= \sum_{j=1}^{K}(w)_{aj}^{(t)}\left(\sum_{i=1}^{3}(H_i^TH_i) + \lambda_1 I\right)_{jb} \\ &\ge w_{ab}^{(t)}\left(\sum_{i=1}^{3}(H_i^TH_i) + \lambda_1 I\right)_{bb} \end{aligned} \tag{15}$$

Thus, Eq. (14) holds and $G(w, w_{ab}^{(t)}) \ge F_{ab}(w)$. We can now demonstrate the convergence of Theorem 1.

Proof of Theorem 1: Replacing $G(w, w_{ab}^{(t)})$ in Eq. (9) by Eq. (12) results in the update rules:

$$\begin{aligned} w_{ab}^{(t+1)} &= w_{ab}^{(t)} - w_{ab}^{(t)}\frac{F'_{ab}(w_{ab}^{(t)})}{2\left(W\sum_{i=1}^{3}\left(H_i^TH_i\right) + \lambda_1 W\right)_{ab}} \\ &= w_{ab}^{(t)}\frac{\sum_{i=1}^{3}\left(X_iH_i\right)_{ab}}{\left(W\sum_{i=1}^{3}\left(H_i^TH_i\right) + \lambda_1 W\right)_{ab}} \\ &= w_{ab}^{(t)}\frac{(X_1H_1 + X_2H_2 + X_3H_3)_{ab}}{(WH_1^TH_1 + WH_2^TH_2 + WH_3^TH_3 + \gamma_1 W)_{ab}} \end{aligned} \tag{16}$$

Since Eq. (12) is an auxiliary function, F_{ab} is non-increasing under this update rule.

S3. Parameter selection

In this section, we discuss how to solve the optimization problem of CeModule to determine the parameter values of our model. As aforementioned, the objective function is not convex on W, H_1, H_2 and H_3, and we cannot analytically compute general solutions. Therefore, an iterative update algorithm is employed to find local minimum of the problem by updating W and H_i iteratively. This iterative procedure converges to the local minima because of the fact that the objective function is bounded below, and the sequence of function values is monotonically decreasing, and the gradients at the convergence are zeros. As the problem is non-convex, we perform the learning process several times with different parameter combinations. The optimum combination is obtained from the following values: α, λ_1, λ_2, and λ_3 are chose from $\{0, 10^{-4}, 10^{-3}, 10^{-2}, 10^{-1}\}$, γ_1 and γ_2 are selected from $\{10^{-2}, 10^{-1}, 0, 5, 10, 20\}$. After repeating this process several times (500 iterations in our experiments), we select the parameter combination from the result that leads to the lowest value for our objective function. Finally, we set α, λ_1, λ_2, λ_3 γ_1 and γ_2 to 0.0001, 0.01, 0.01, 0.01, 10, and 10, respectively.

For the sub-space dimension K, we set K to 70 on the basis of a miRNA cluster analysis. Parameter T is a given threshold that is employed to determine the module members (lncRNAs/miRNAs/ mRNAs). In this way, the number of lncRNAs/miRNAs/mRNAs in each identified module strongly relies on the value of T. Too large or too small value of the threshold T may generate inappropriate modules. We expect that the pair-wise lncRNAs (miRNAs or mRNAs) within each module identified by CeModule are highly correlated, and then adopt the average absolute Pearson correlation coefficient (PCC) of all modules, namely $\frac{1}{K}\sum_{i}^{K}\left(2\sum_{i}|corr(x,y)|\Big/(M_i*(M_i-1))\right)$, to determine the value of T for lncRNAs/miRNAs/mRNAs based on the expression profiles, where *corr* is a function that calculates PCC for the pair (x, y), and x and y represent a pair of lncRNAs (miRNAs or mRNAs) in module i, K and M_i denote the number of modules and the number of lncRNAs (miRNAs or mRNAs) in module i, respectively. After conducting a series of tests based on our experiment scenario, we finally set T=5/3/4 (5/3/3) for selecting lncRNAs/miRNAs/mRNAs, and obtained 70 modules with an average of 68.2 (46.1) lncRNAs, 6.3 (5.5) miRNAs, and 55.5 (48.1) mRNAs per module for OV (UCEC) dataset

S4. Kaplan-Meier survival analysis to identify clinically related modules

Here, for instance, we also investigated whether the modules identified by CeModule based on OV dataset were associated with the survival of ovarian cancer patients, and a Kaplan-Meier (KM) survival analysis was performed using the R package survival. The clinical data are downloaded from TCGA, and 383 samples are retained after removing those not included in the expression data or those with unavailable survival time. For each module, we classified the patients into two groups ("low group" vs. "high group") according to the lower or higher module-averaged lncRNA expression levels than the sample means. The log-rank test was employed to evaluate the statistical significance for each module, and then the p-values are further adjusted by multiple test correction using Benjamini-Hochberg method (FDR<0.05).

The prediction of survival power of a module is better than that of an individual gene. As shown in Figure S5, we found that a representative prognostic modules (module 43) in ovarian cancer could significantly separate patients into two groups with different clinical outcomes. The module 43 was significantly associated with the survival of OV patients (FDR=2.35e-02), and the median survival times of the "high group" and "low group" are 1583 and 1264 days, respectively. Notably, the patients of the "low group" faced higher risks as shown in Figure S5. The observations imply that the proposed method has a potential ability to discover modules that could provide useful information for the prediction of cancer prognosis.

S5. Supplementary figures

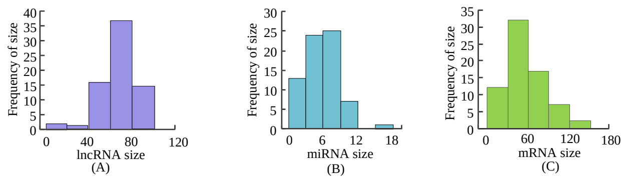

Figure S1. Topological features of the identified modules and the ceRNA regulatory module network. The distributions of number of **(A)** lncRNAs, **(B)** miRNAs, and **(C)** mRNAs for the identified modules in OV dataset.

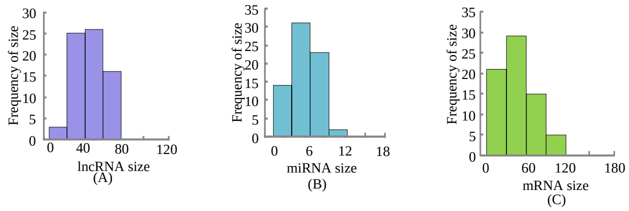

Figure S2. Topological features of the identified modules and the ceRNA regulatory module network. The distributions of number of **(A)** lncRNAs, **(B)** miRNAs, and **(C)** mRNAs for the identified modules in UCEC dataset.

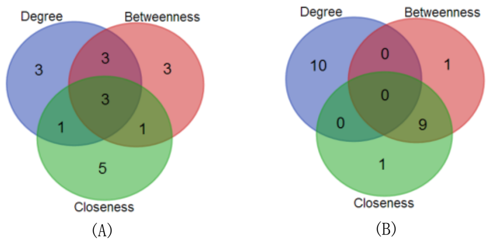

Figure S3. Overlap of the top 10 (A) miRNAs and (B) mRNAs across three dimensions (degree, betweenness centrality, and closeness centrality) in OV dataset.

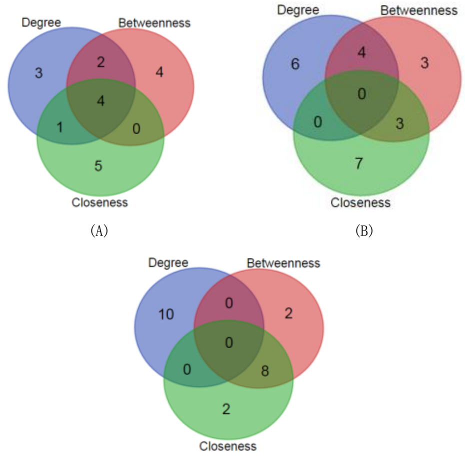

Figure S4. Overlap of the top 10 (A) lncRNAs, (B) miRNAs and (C) mRNAs across three dimensions (degree, betweenness centrality, and closeness centrality) in UCEC dataset.

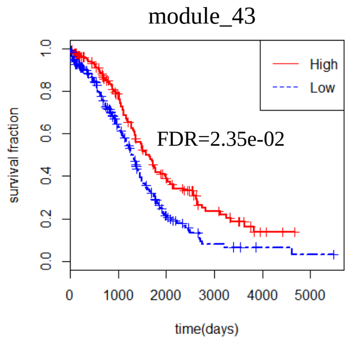

Figure S5. Kaplan-Meier survival curves for ovarian cancer patients classified into two groups using the module-averaged lncRNA expression levels.

Supplementary Table includes:

Table S1. The list of all the identified regulatory modules that involving lncRNAs, miRNAs and mRNAs.
Please refer to file Table_S1_module_list.xlsx

Table S2. The top 10 lncRNAs, miRNAs and mRNAs with the highest degree, closeness centrality, and betweenness centrality in UCEC.

Rank	Degree			Betweenness			Closeness		
	lncRNAs	miRNAs	mRNAs	lncRNAs	miRNAs	mRNAs	lncRNAs	miRNAs	mRNAs
1	XIST	let-7b	CORO1A	GAS5	mir-155	RPL24	UBXN8	mir-155	TUBG1
2	SCAMP1	mir-93	RPS14	DCP1A	mir-296	ZNF664	AC093838.4	mir-296	CDC5L
3	MALAT1	mir-141	NCF4	NEAT1	mir-532	PKP3	XIST	mir-106b	PKP3
4	MAL2	mir-10a	RPS4X	UBXN8	mir-16	EVL	MIR155HG	mir-301a	HMGCL
5	C17orf76-AS1	mir-200b	RPS9	XIST	let-7b	SYNGR3	RP11-220I1.1	mir-378c	EVL
6	DCP1A	mir-34a	RPSA	RP11-220I1.1	mir-93	UBE2D1	MALAT1	mir-425	UBE2D1
7	C11orf95	mir-590	CD53	SNHG12	mir-10a	TUBG1	TUBA4B	mir-935	ZNF664
8	UBXN8	mir-200a	CD37	SNHG1	mir-34a	HMGCL	TUBBP1	mir-940	SYNGR3
9	RP11-220I1.1	mir-15b	RPL5	MALAT1	mir-29b	ZFHX3	SCAMP1	mir-96	ACSL3
10	SEC22B	mir-18a	CD48	MAL2	mir-30c	CDC5L	HCP5	mir-532	ELOVL5

Table S3. Results of the enriched GO biological processes for the identified modules.
Please refer to file Table_S3_GO.xlsx

Table S4 Results of the enriched KEGG pathways for the identified modules.
Please refer to file Table_S4_KEGG.xlsx

Table S5. The list of regulatory modules enriched in miRNA cluster and miRNA family.
Please refer to file Table_S5_miRNA_cluster_family.xlsx

Table S6. Known OV/UCEC-related lncRNAs/miRNAs/mRNAs and cancer-related lncRNAs/miRNAs/mRNAs in modules.
Please refer to file Table_S6_cancer_modules.xlsx

Table S7. Differentially expressed miRNAs identified in modules.
Please refer to file Table_S7_DE_modules.xlsx
